# Orthostatic Changes in Hemodynamics and Cardiovascular Biomarkers in Dysautonomic Patients

**DOI:** 10.1371/journal.pone.0128962

**Published:** 2015-06-08

**Authors:** David Nilsson, Richard Sutton, Widet Tas, Philippe Burri, Olle Melander, Artur Fedorowski

**Affiliations:** 1 Department of Clinical Sciences, Lund University, Clinical Physiology and Nuclear Medicine Unit, Skåne University Hospital, Malmö, Sweden; 2 National Heart and Lung Institute, Imperial College, St Mary's Hospital Campus, London, United Kingdom; 3 Department of Clinical Sciences, Lund University, Hypertension and Cardiovascular Disease Group, Clinical Research Centre, Skåne University Hospital, Malmö, Sweden; 4 Department of Cardiology, Skåne University Hospital, Malmö, Sweden; Uppsala University, SWEDEN

## Abstract

**Background:**

Impaired autonomic control of postural homeostasis results in orthostatic intolerance. However, the role of neurohormones in orthostatic intolerance has not been explained.

**Methods:**

Six-hundred-and-seventy-one patients (299 males; 55±22 years) with unexplained syncope underwent head-up tilt (HUT) with serial blood sampling. Systolic blood pressure (SBP) and heart rate (HR) supine, after 3min, and lowest BP/highest HR during HUT were recorded. Plasma levels of epinephrine, norepinephrine, renin, C-terminal-pro-arginine-vasopressin (CT-proAVP), C-terminal- endothelin-1 (CT-proET-1), and mid-regional-fragment of pro-atrial-natriuretic-peptide (MR-proANP) were determined at supine and 3min of HUT. Multivariate-adjusted logistic regression model was applied to compare 1^st^ (reference) with 4^th^ quartile of 3 min and maximal ΔSBP/ΔHR (i.e. pronounced hypotension or tachycardia) vs. changes in neuroendocrine biomarkers, respectively.

**Results:**

Higher resting CT-proET-1 predicted BP fall at 3min (Odds ratio (OR) per 1 SD: 1.62, 95%CI 1.18–2.22; p = 0.003), and max BP fall during HUT (1.82, 1.28–2.61; p = 0.001). Higher resting CT-proAVP predicted BP fall at 3min (1.33, 1.03–1.73; p = 0.03), which was also associated with increase in CT-proAVP (1.86, 1.38–2.51; p = 0.00005) and epinephrine (1.47, 1.12–1.92; p = 0.05) during HUT. Lower resting MR-proANP predicted tachycardia at 3min (0.37, 0.24–0.59; p = 0.00003), and max tachycardia during HUT (0.47, 0.29–0.77; p = 0.002). Further, tachycardia during HUT was associated with increase in epinephrine (1.60, 1.15–2.21; p = 0.005), and norepinephrine (1.87, 1.38–2.53; p = 0.005).

**Conclusions:**

Resting CT-proET-1 and CT-proAVP are increased in orthostatic hypotension, while resting MR-proANP is decreased in postural tachycardia. Moreover, early BP fall during orthostasis evokes increase in CT-proAVP and epinephrine, while postural tachycardia is associated with increase in norepinephrine and epinephrine.

## Introduction

“Orthostatic intolerance” refers to a group of clinical conditions in which symptoms induced by upright posture are ameliorated by recumbency[1]. On standing, the gravitational volume shift causes a redistribution of circulating blood by pooling within the capacitance vessels below the diaphragm [2]. A normal hemodynamic response to changes in posture requires normal function of the cardiovascular, endocrine, and autonomic nervous systems [3]. In cardiovascular dysautonomic states, the circulatory redistribution may lead to hypotension and/or tachycardia, thus compromising cerebral blood flow with symptoms such as blurred vision, fatigue, dizziness, and, in the most extreme cases, syncope[4].

Two main variants of orthostatic intolerance, orthostatic hypotension (OH) and postural tachycardia syndrome (POTS), distinctly differ from syncope due to classical vasovagal reflex[5]. In classical reflex syncope, cardiovascular reflexes become transiently inappropriate while in orthostatic hypotension (OH) sympathetic efferent activity may be chronically impaired. The etiology of POTS, which shares features of a chronic disorder with OH, remains under debate.

Some studies have indicated that neuropeptides may play an important role in cardiovascular dysautonomia and syncope [6]. We have previously reported that suppression of atrial natriuretic peptide is associated with vasovagal reflex syncope, whereas increased endothelin-1 can be found in OH [7]. Further, a study measuring circulating vasopressin and epinephrine during head-up tilt test (HUT) has shown that these neurohormones are elevated prior to reflex syncope, while norepinephrine changes little [8].

The aim of this study was to explore how circulating levels of the most important neurohormones involved in cardiovascular homeostasis, both at rest prior to HUT and during its early phase relate to the well-documented hemodynamic responses to HUT. As most of cardiovascular neurohormones, in particular atrial natriuretic peptide, endothelin-1, and vasopressin, are characterized by a short half-life of a few minutes, we applied newly developed laboratory assays to detect their stable fragments, thus allowing better estimation of neurohormone biosynthesis[9–11]. Patients were those referred for investigation of syncope and were considered to warrant better understanding of the mechanisms involved. Consequently, our study was observational with no attempt to include a control group. We hypothesized that hemodynamic changes induced by HUT should be correlated with neuroendocrine changes permitting targeted therapy in dysautonomic patients.

## Materials and Methods

### Study setting and population

Between August 2008 and October 2013, a total of 836 patients with suspected syncope i.e. TLOC unexplained by initial evaluation [4], were investigated at the Syncope Unit of Skåne University Hospital. Of these, 671 patients (299 males and 372 females; age, 55 ± 22 years; range, 15–93) underwent HUT according to the Italian protocol [12] and accepted blood sampling during the test. Patients were recruited through referrals from primary care and from hospitals in the southern region of Sweden. Prior to HUT at the Syncope Unit, additional tests may have been carried out, and included resting, exercise and continuous 24-h (Holter) ECG, external and implantable event recorder, echocardiography, coronary angiography, brain imaging, and EEG. Patients with cardiac syncope, neurological, metabolic, and toxic causes of TLOC were not included in the cohort of 671 patients, also those with signs of advanced dementia and physical disability.

### Examination protocol

The patients participating in the study were asked to take their regular medication and fast for two hours before the test, although they were allowed to drink water *ad libitum*. Prior to examination the patients were asked to complete a questionnaire, which explored past medical history, duration, frequency and features of syncope-related symptoms, smoking status, and current pharmacological treatment.

The specially designed HUT protocol included peripheral vein cannulation, supine rest for 10 minutes, blood sampling both at supine rest and in the upright position 3 minutes after elevation of the table at an angle of 60–70°, and optional nitroglycerin provocation according to the Italian protocol [12]. Nitroglycerin (400 μg spray sublingually) was administrated first after 20 min of passive HUT if syncope had not occurred and the hemodynamic parameters were stable that is no significant hypotension (SBP<90 mmHg) or orthostatic intolerance due to sinus tachycardia > 120 bpm were observed. Thus, this nitroglycerin phase played no part in any of the neuroendocrine measurements or in ultimate diagnosis of OH and POTS, and only relates to the ultimate diagnosis of VVS.

Beat-to-beat blood pressure (BP) and electrocardiogram (ECG) were continuously monitored using a noninvasive validated method (Nexfin monitor, BMEYE, The Netherlands) [13], and subsequently analyzed offline using a dedicated program provided by the monitor manufacturer. Mean BP and heart rate (HR) in supine position, after 3 minutes of HUT, and at the lowest BP/highest HR during passive orthostasis were calculated as an average of a 30-second period.

The predefined point for the second hemodynamic assessment and blood sampling assigned to 3min of HUT was selected to comply with the current definition of classical vs. delayed orthostatic hypotension [14], and was also determined by the cut-off point that is widely used for hemodynamic distinction between classical vasovagal syncope and both OH and POTS[4]. At that chosen point there have typically been no hemodynamic changes in patients who go on to show vasovagal syncope whereas those with classical OH and POTS have already demonstrated notable falls in BP (OH) or pronounced rise in heart rate (POTS).

The third assessment of the hemodynamic parameters between 3 and 20min of HUT, corresponding to lowest SBP/highest HR prior to obvious activation of vasovagal reflex and/or syncope or end of the passive HUT, was intended to identify those with delayed hemodynamic instability i.e. delayed OH or progressive postural tachycardia. The onset of vasovagal reflex was identified by typical prodrome and/or an abrupt change in hemodynamic parameters such as bradycardia and/or pronounced hypotension. Accordingly, the timing of the third hemodynamic assessment was selected by visual analysis of SBP and HR curves, and by consensus between two study investigators (DN and AF). If the maximal BP fall occurred before 3min of HUT and led to syncope and/or test termination, the authors decided to assign the corresponding hemodynamic parameters to both the second (3min) and third measurement (lowest SBP).

The Regional Ethical Review Board in Lund, Sweden accepted the study protocol (ref no 82/2008), and all study participants gave their written informed consent. Written consent on behalf of the minors/children was obtained by from parents.

### Neuroendocrine biomarkers

**Fig 1:**
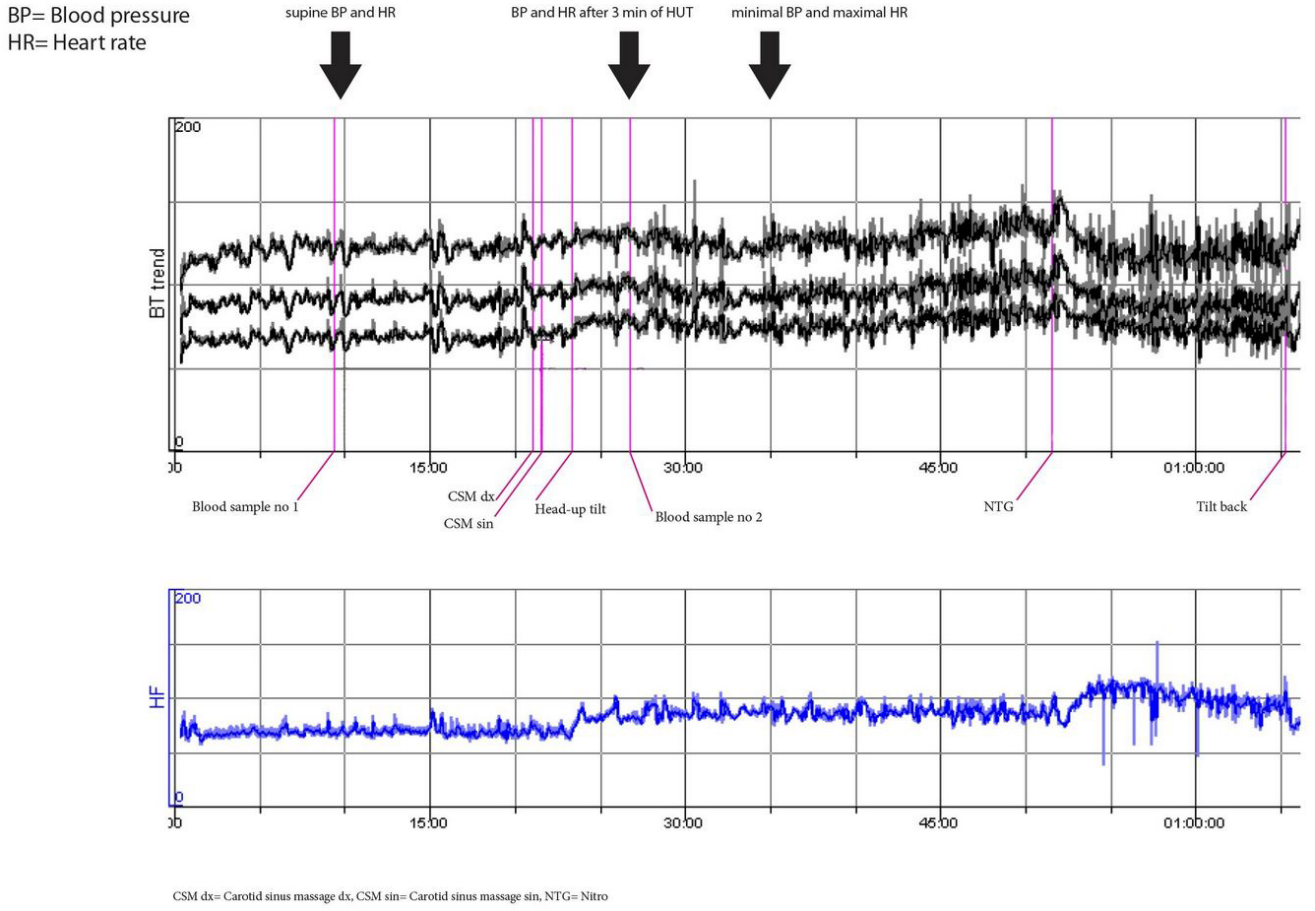
Normal hemodynamic response to passive head-up tilt test and nitroglycerin (woman, 32 years). This patient is representative of lowest (reference) quartiles of both ΔSBP and ΔHR (i.e. Q1).

**Fig 2:**
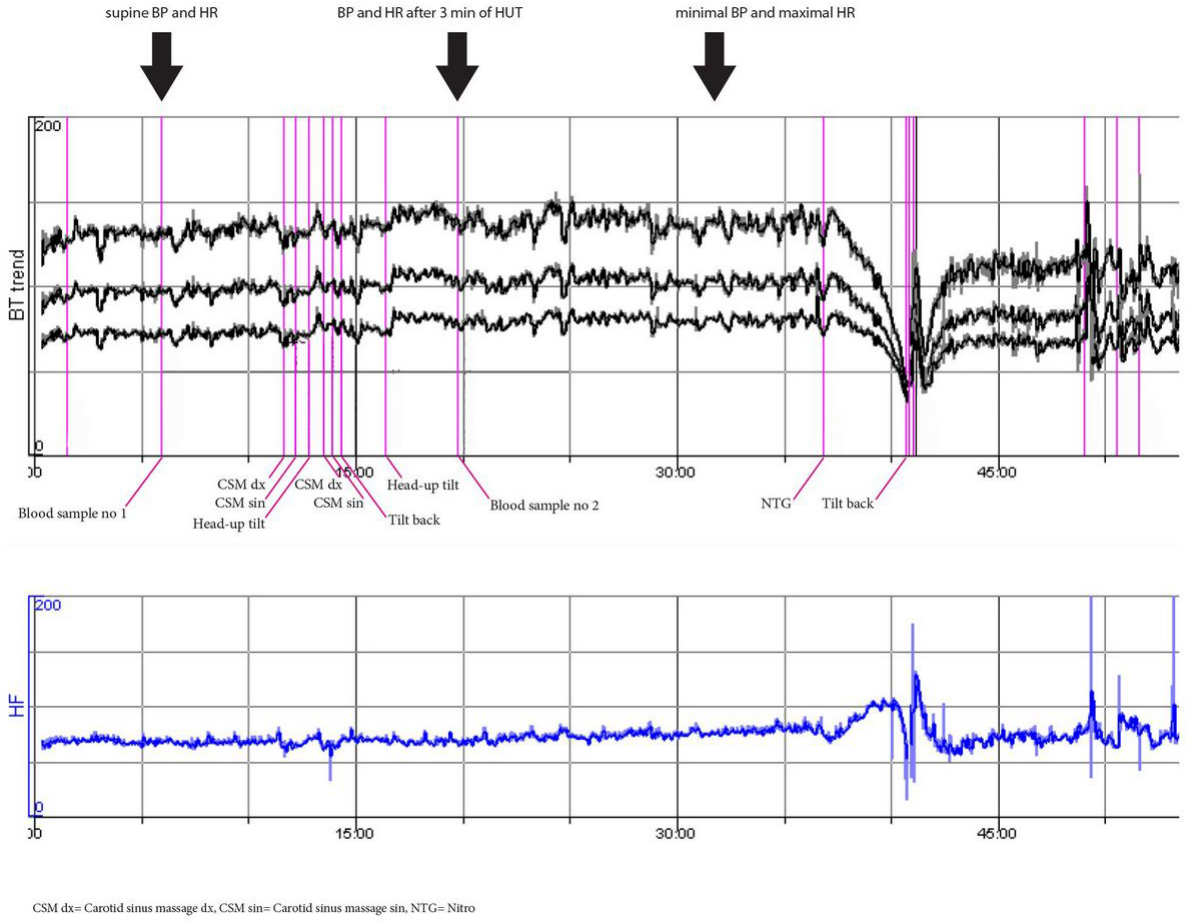
Normal hemodynamic response to passive head-up tilt test and vasovagal reflex induced by nitroglycerine (man, 50 years). This patient is representative of lowest (reference) quartiles of both ΔSBP and ΔHR (i.e. Q1).

**Fig 3:**
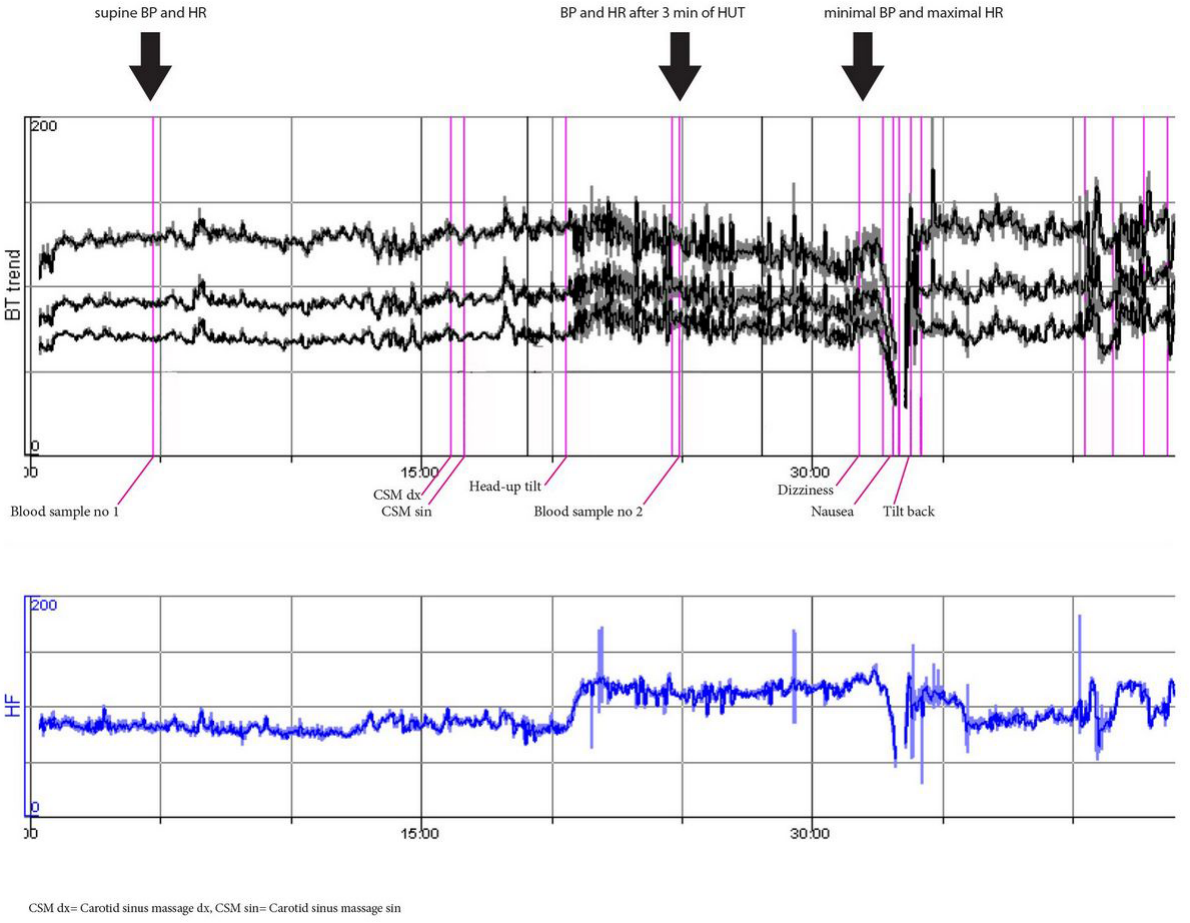
Pronounced orthostatic tachycardia (≈POTS) and vasovagal reflex syncope (woman, 24 years). This patient is representative of highest quartiles of ΔHR (i.e. Q4) both after 3 min of HUT and at the maximal heart rate during HUT.

**Fig 4:**
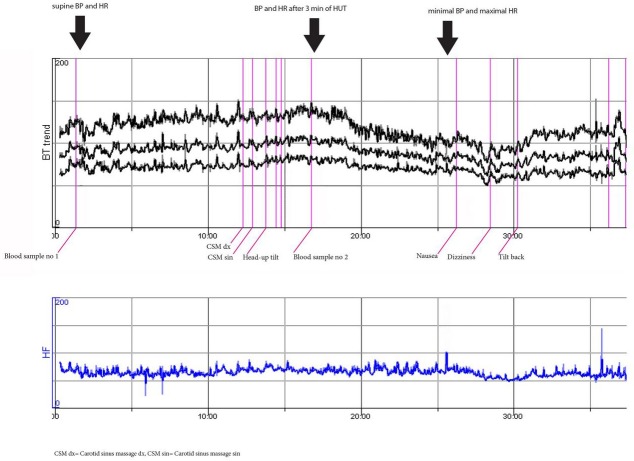
Delayed orthostatic hypotension and syncope probably due to vasovagal reflex activation (woman, 53 years). This patient is representative of highest quartile of max ΔSBP (i.e. Q4) at the minimal SBP during HUT but not after 3 min of HUT.

**Fig 5:**
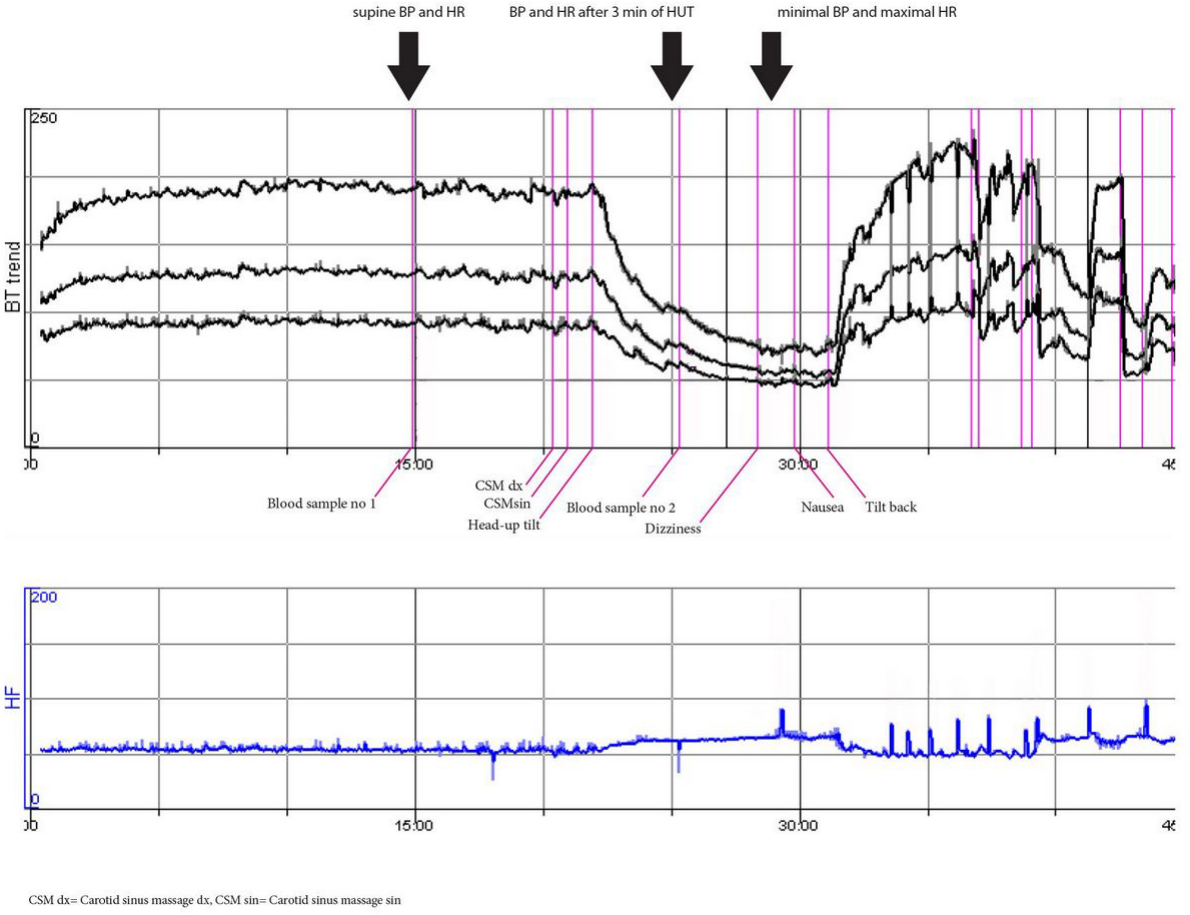
Pronounced classical orthostatic hypotension and syncope (man, 65 years). This patient is representative of highest quartiles of ΔSBP (i.e. Q4) both after 3 min of HUT and at the minimal SBP during HUT.

Blood samples during supine rest before HUT and after 3min of HUT (Figs 1–5) were used for determination of plasma levels of six cardiovascular neurohormones with an established systemic effect on circulation: epinephrine, norepinephrine, renin, C-terminal-pro-arginine-vasopressin (CT-proAVP), C-terminal- endothelin-1 (CT-proET-1), and mid-regional-fragment of pro-atrial-natriuretic-peptide (MR-proANP). Plasma biomarkers were measured from blood samples (16x250 μl aliquots of EDTA plasma in plastic thermotubes) that had been frozen at -80° C after collection. CT-proAVP, CT-proET-1, and MR-proANP were measured using the following assays according to the manufacturer’s instructions: Thermo Scientific BRAHMS CT-proAVP KRYPTOR, Thermo Scientific BRAHMS CT-proET-1 KRYPTOR, and Thermo Scientific BRAHMS MR-proANP KRYPTOR (BRAHMS GmbH, part of Thermo Fisher Scientific, Neuendorfstraße 25, 16761 Hennigsdorf, Germany). Concentrations of epinephrine and norepinephrine were determined by high-performance liquid chromatography with fluorescence detection[15]. Plasma renin concentrations were analyzed using an immunoradiometric assay (Renin III Generation; Cisbio Bioassays International, 30200 Codolet, France). The total amount of blood drawn for the analyses was 60ml (30+30ml), and no fluid substitution was given.

### Statistical analyses

The main characteristics of study population are presented as mean and standard deviation for continuous variables, and percentages for categorical variables. Study population was stratified by quartiles of four HUT-related hemodynamic parameters, ΔSBP_3min_ (SBP_supine_−SBP_HUT 3min_), ΔSBP_maximal_ (SBP_supine_−SBP_HUT minimal_), ΔHR_3min_ (HR_HUT 3min_−HR_supine_), and ΔHR_maximal_ (HR_HUT maximal_−HR_supine_). As the assumption of normal distribution was not satisfied, interquartile differences in neurohormone concentrations (i.e. resting levels and change after 3 min of HUT, respectively) were analyzed using Kruskal-Wallis tests, and corresponding neurohormone concentrations were reported as median + interquartile range. Next, multivariate-adjusted logistic regression models (incl. age and gender as covariates) were applied by entering 4^th^ quartile (Q4, maximal deviation from baseline) vs. 1^st^ quartile (Q1, reference) of the assessed hemodynamic parameter as the categorical dependent variable, and standardized plasma concentration of the analyzed neurohormone (presented as standard scores) in supine position or its change after 3min of HUT as the independent variable, respectively.

As concentrations of the analyzed neurohormones were significantly right-skewed, we performed log-transformation prior to analyses. This model was designed to analyze concentrations of neurohormones in the subset of patients with the most pronounced hemodynamic divergence from baseline (i.e. hypotension or tachycardia) irrespective of whether the formal diagnostic criteria of OH and POTS were met or not during HUT. Accordingly, the final HUT diagnosis was not the object of analysis as the focus of this study was the presyncopal and not syncopal phase of the test. All analyses were performed using IBM SPSS Statistics version 22 (SPSS Inc., Chicago, IL). All tests were two-sided, whereby *p*<0.05 was considered statistically significant.

## Results

### Plasma concentrations of neuroendocrine biomarkers vs. quartiles of SBP and heart rate change during HUT

Summary of biometric and hemodynamic parameters for the whole study population is presented in Table 1. There were significant diversities in neuroendocrine characteristics depending on how the circulatory system responded to HUT (see Supplementary Tables 1–4). Supine levels of MR-proANP, CT-proET-1, CT-proAVP, renin, and norepinephrine (maximal SBP decrease only) were higher among those who demonstrated pronounced fall in SBP during HUT. In contrast, those patients who responded with a distinct HR increase during HUT, displayed lower supine levels of MR-proANP, CT-proET-1, CT-proAVP, epinephrine, and norepinephrine, whereas the latter two rose significantly on standing. It should be emphasized, however, that not all patients in the highest quartiles of ΔHR met the conventional diagnostic criteria of POTS (i.e. increase over 30 beats/min or tachycardia over 120 beats/min on standing associated with symptom of orthostatic intolerance) but all of them demonstrated an excessive increase in heart frequency. More specifically, in the highest quartiles, mean HR increase was 25 beats/min (range, 16–63) for 3min of HUT, and 34 beats/min (range, 22–66) for maximal HR recorded during HUT. Thus, the 4^th^ (highest) quartiles of ΔHR represented a subset of patients with exaggerated chronotropic response to passive orthostasis, while median values of ΔHR for the entire study population (+9 and +11 beats/min, respectively) were very similar to those observed in non-syncopal adults [16].

**Table 1:** Clinical characteristics of study population (n = 671) including haemodynamic parameters observed during head-up tilt test.

Age (years)	55±22
Gender (male, %)	44.6
Systolic BP supine	134±22 mmHg
Systolic BP standing 3 min	130±24 mmHg
Min systolic BP	112±25 mmHg
Diastolic BP supine	72±9 mmHg
Diastolic BP standing 3 min	76±12 mmHg
Min diastolic BP	69±13 mmHg
Heart rate supine	69±11 beats/min
Heart rate standing 3 min	79±15 beats/min
Heart rate standing max	83±17 beats/min

All values are given as mean ± standard deviation, unless otherwise stated.

### Plasma concentrations of neuroendocrine biomarkers in normal vs. altered hemodynamic responses to HUT

The following paragraph refers to logistic regression models comparing highest (a tendency to either orthostatic hypotension or postural tachycardia) with lowest quartiles of the assessed hemodynamic responses to HUT as a reference (Supportive Information S1–S4 Tables).

### CT-proET-1

**Fig 6:**
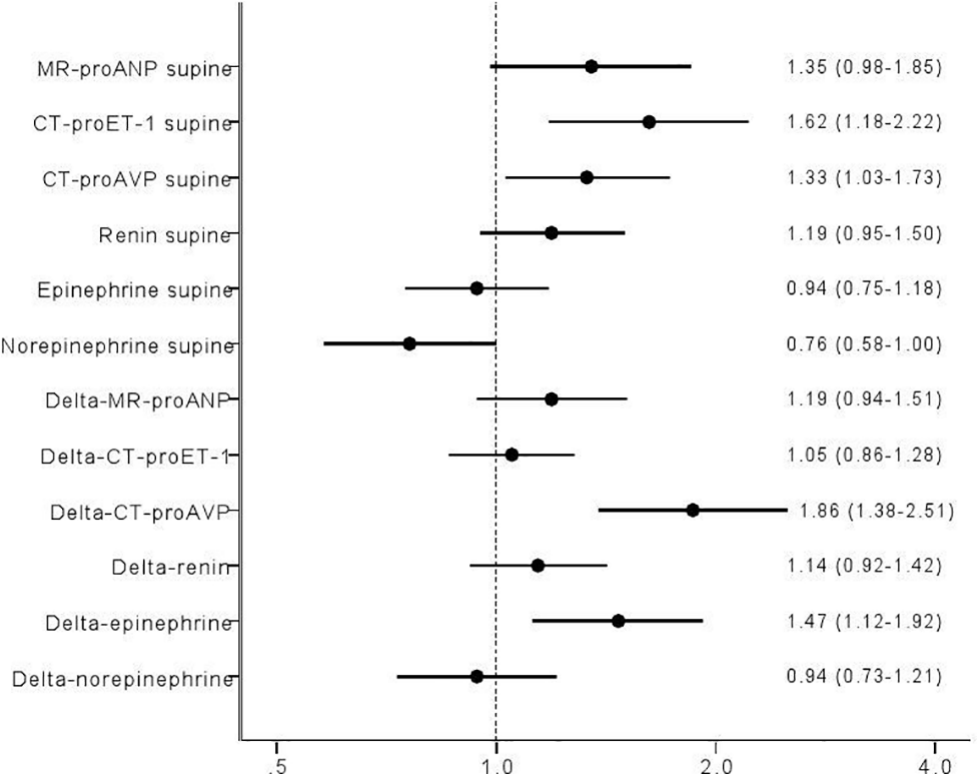
ORs log regression Q4 vs Q1 adj age sex delta SBP 3min log transformed.

**Fig 7:**
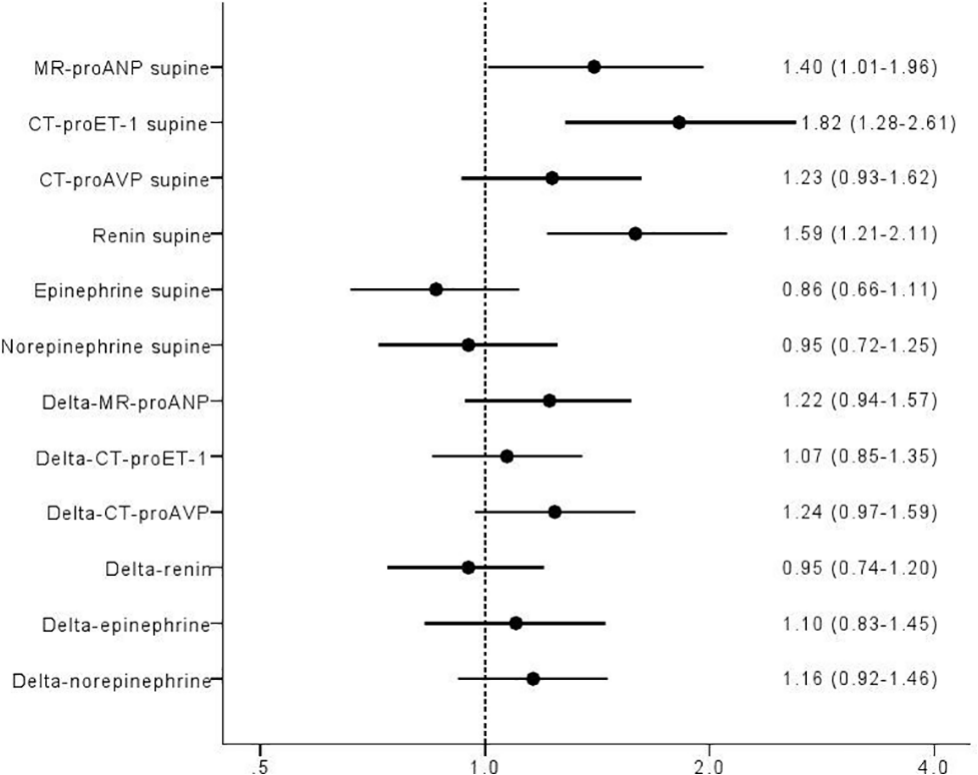
ORs log regression Q4 vs Q1 adj age sex delta SBP max log transformed.

Higher resting levels of CT-proET-1 predicted decrease in SBP both at 3min (Fig 6; OR per 1 SD: 1.62, 95% CI 1.18–2.22; p = 0.003), and max SBP decrease during HUT (Fig 7; OR per 1 SD: 1.82, 95% CI 1.28–2.61; p = 0.001).

### MR-proANP

**Fig 8:**
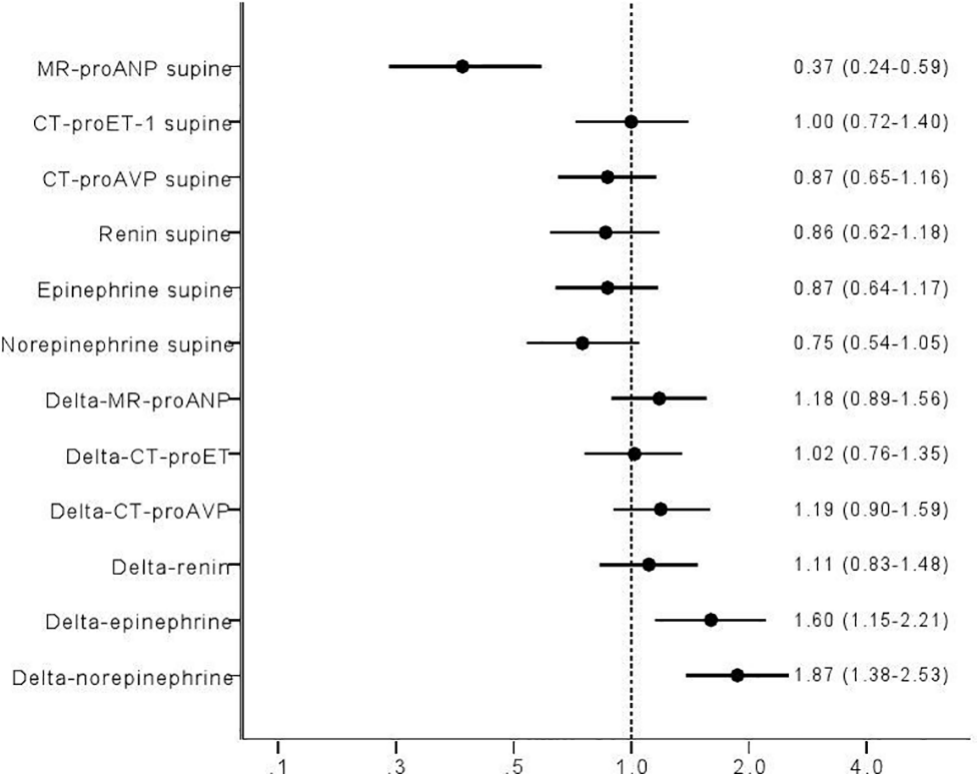
ORs log regression Q4 vs Q1 adj age sex delta HR 3 min log transformed.

**Fig 9:**
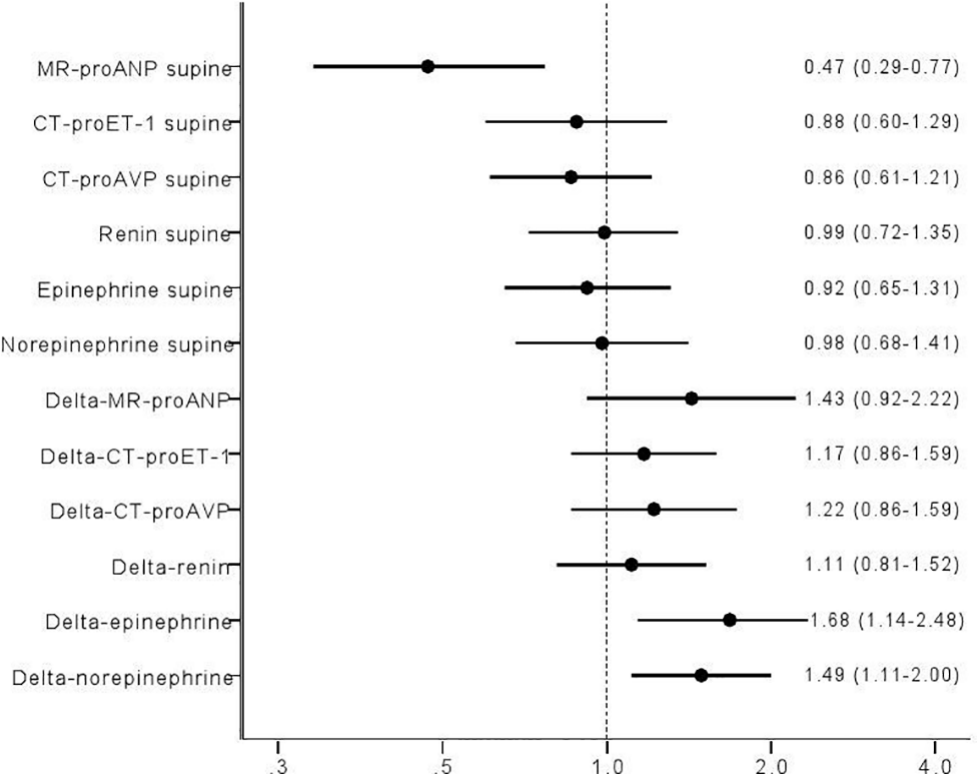
ORs log regression Q4 vs Q1 adj age sex delta HR max log transformed.

Higher resting levels of MR- proANP predicted max SBP decrease (Fig 7; Odds ratio (OR) per 1 SD: 1.40, 95% CI 1.01–1.96; p = 0.05), whereas lower resting MR-proANP predicted increase in HR both at 3min (Fig 8; OR per 1 SD: 0.37, 95% CI 0.24–0.59; p = 0.00003), and max HR increase during HUT (Fig 9; OR per 1 SD: 0.47, 95% CI 0.29–0.77; p = 0.002).

### CT-proAVP

Higher resting levels of CT-proAVP predicted decrease in SBP at 3min HUT (Fig 6; OR per 1 SD: 1.33, 95% CI 1.03–1.73; p = 0.03) but not max SBP decrease. Decrease in SBP at 3min paralleled increase in CT-proAVP (Fig 6; OR per 1 SD: 1.86, 95% CI 1.38–2.51; p = 0.00005).

### Renin

Higher resting levels of renin predicted max SBP decrease (OR per 1 SD: 1.59, 95% CI 1.21–2.11; p = 0.001) but not at 3min of HUT (Figs 6–7).

### Epinephrine

Decrease in SBP at 3min (Fig 6; OR per 1 SD: 1.47, 95% CI 1.12–1.92; p = 0.05) paralleled increase in epinephrine after 3min. Further, increase in epinephrine paralleled both maximal and 3min increase in HR (Figs 8–9; OR per 1 SD: 1.68, 95% CI 1.14–2.48; p = 0.009, and 1.60, 95% CI 1.15–2.21; p = 0.005, respectively).

### Norepinephrine

Lower resting levels of norepinephrine predicted decrease in SBP at 3min HUT (Fig 6; OR per 1 SD: 0.76, 95% CI 0.58–1.00; p = 0.05). In contrast, norepinephrine increment paralleled both maximal and 3min increase in HR (Figs 8–9; OR per 1 SD: 1.49, 95% CI 1.11–2.00; p = 0.009, and 1.87, 95% CI 1.38–2.53; p = 0.005, respectively).

Further adjustments for antihypertensive treatment (n = 127; both total and by separate classes) and exclusion of patients (n = 27) with prevalent left ventricle ejection fraction (LVEF) <55% did not substantially change our results, except for MR-proANP in prediction of max SBP decrease, which was non-significant after exclusion of LVEF<55% (OR per 1 SD: 1.36, 95% CI 0.97–1.90; p = 0.07).

## Discussion

### Main findings

This study provides data from a large series of patients with a history of syncope and elucidates the neuroendocrine pattern associated with dysautonomic responses to orthostasis. We have observed that resting CT-proET-1, CT-proAVP, MR-proANP, and renin are elevated in patients with orthostatic hypotension, of these, two latter in the delayed form only, while BP fall on standing is associated with a significant release of vasopressin and epinephrine. In contrast, in patients with postural tachycardia, resting MR-proANP is downregulated, whereas both epinephrine and norepinephrine significantly increase on standing.

Previous studies have examined neurohormones during or after syncope, paying little or no attention to hemodynamic changes preceding syncope, which might explain the contradictory findings observed in small series. On the contrary, the present study investigated the pre-test neuroendocrine profile and its early changes during HUT in relation to hemodynamic responses (early and late during HUT) offering insights into the mechanisms of cardiovascular dysautonomia in syncope.

### CT-proET-1

Increased CT-proET-1 baseline levels are associated with orthostatic hypotension [7] but elevated ET-1 has also been reported in patients with vasovagal syncope [23,24]. However, it is important to note that presyncopal phase during HUT has not been routinely assessed in previous studies and an important subgroup of patients with ultimate reflex syncope might have underlying orthostatic intolerance that has not been noted. Therefore, although apparently contradictory, previous results and ours may not be directly comparable.

This study demonstrated that higher resting levels of CT-proET-1 consistently predicted decrease in SBP during the entire HUT, which suggests that CT-proET-1 might be a promising predictor of orthostatic hypotension, possibly of both classical and delayed forms.

### MR-proANP

Suppression of ANP production has been found to be associated with reflex syncope [7]. Supine levels of ANP have also been reported to be lower in patients with vasovagal syncope than in patients with neurogenic OH [17], and to be higher in cardioinhibitory than in vasodepressor reflex types at baseline [18]. It has also been observed that a decrease of ANP occurs during prolonged orthostatic stress [19]. The most important factor triggering ANP secretion is thought to be mechanical stretching of the atria which takes place when the extracellular fluid volume or blood volume is elevated [20] but can also be stimulated by paracrine factors such as ET-1 [20]. Interestingly, higher levels of circulating ANP and lower SBP have been reported to be associated with the same gene variants [21].

It is tempting to speculate that the lower supine levels of ANP seen in patients with orthostatic tachycardia may be due to the lower blood volume and thus absence of mechanical stretching of the atria whereas higher resting levels of ANP could predict decrease in blood pressure due to stimulation of ANP secretion by neurohormones such as for example ET-1 or even by BP-associated gene variants, or by supine hypertension, often seen in OH[22].

### CT-proAVP

Previous studies have reported significantly higher levels of AVP at baseline in those with VVS compared with non–responders [8]. Moreover, earlier studies have shown that AVP increases both in VVS and HUT-negative patients on standing [25]. Increase in AVP during HUT has also been reported in both subjects with cardiogenic syncope and non-syncopal subjects, however, being more pronounced in patients experiencing syncope but without any differences between the groups at rest [26]. Other studies have demonstrated similar results, i.e. an increase in AVP during HUT [23,27,28], being more marked in patients with syncope.

This study demonstrated that higher resting level of CT-proAVP and pronounced AVP release during HUT paralleled orthostatic BP fall. These results suggest a possible compensatory role for AVP in patients with both classic orthostatic hypotension and early vasodepressor VVS, where increase in AVP during HUT may act as an important rescue mechanism invoked by cerebral hypoperfusion and/or falling left ventricle/right atrial filling pressure detected by mechanoreceptors.

### Renin

A previous study has demonstrated that 25% of normal subjects who develop vasovagal syncope after upright tilt have reduced renin activity both in magnitude and duration compared with the normal response [29]. Another study has shown increased renin levels at presyncope in healthy subjects [30]. Moreover, unlike patients with cardioinhibitory syncope, the renin-angiotensin-aldosterone axis has been reported to be activated in patients with vasodepressor syncope [31].

Our data demonstrate that higher resting levels of renin predicts maximal decrease in SBP occurring first after the initial 3min of HUT, which suggests an elevated renin activity to predict delayed orthostatic hypotension.

### Epinephrine

A pronounced elevation of plasma epinephrine has been reported in patients with vasovagal syncope, and has been thought to play a role by possible vasodilatation resulting in hypotension [6]. A significant increase in epinephrine levels has also been observed immediately after standing, with a constant rise during prolonged orthostatic stress [19]. Moreover, baseline plasma epinephrine levels have been shown to be higher in patients with syncope compared with non-syncopal controls, and the same pattern has been observed during HUT [8]. In parallel, patients with a cardioinhibitory response have been reported to have a steep rise in plasma epinephrine at syncope [18].

As demonstrated in this study, decrease in SBP at 3min paralleled increase in epinephrine, suggesting that epinephrine could act as a trigger- or, alternatively, as a compensatory mechanism in the acute phase of orthostatic hypotension (i.e. classical) but not after prolonged standing (i.e. delayed). Further, both 3min and maximal increase in HR during HUT were associated with increase in epinephrine suggesting an important role of this neurohormone in orthostatic tachycardia.

### Norepinephrine

Norepinephrine has been shown to rise with acute orthostatic BP fall [19]. Patients with autonomic failure have been found to have lower values of norepinephrine when supine and a near 10-fold less rise during orthostatic stress when compared with vasovagal syncope and normal controls, whereas patients with vasovagal syncope have lower levels of supine noradrenaline but a more pronounced increase during the first minutes of HUT [17]. It has been shown that no significant differences in norepinephrine between HUT positive and negative groups occurred at baseline, although both showed a rising trend during HUT (7). Moreover, a study of age-matched syncope patients found that those with a cardioinhibitory response were characterized by activation of the sympathetic system, evidenced by a rise in plasma norepinephrine [18].

This study demonstrated that lower resting levels of norepinephrine predicted decrease in SBP at 3min HUT, and thus may predict early SBP fall as in classical orthostatic hypotension or, alternatively, in the sudden-onset vasodepressor reflex. Moreover, increase in norepinephrine after 3min of HUT paralleled both early and maximal increase in HR during HUT. As for epinephrine (see above), norepinephrine may have an important role in orthostatic tachycardia. Consequently, the possible involvement in POTS of both norepinephrine and epinephrine has to be further evaluated.

### Final remarks

An effective therapy against cardiovascular dysautonomic syndromes such as orthostatic hypotension and POTS is not available today. Although both heart rate control and vasoactive drugs have been tested and are still being used *ex iuvantibus*, convincing data and international consensus on their efficacy are lacking. Therefore, targeting the main neuroendocrine systems involved in circulatory homeostasis, which demonstrate a distinct alteration in the main forms of orthostatic intolerance, seems to be a promising strategy. Pharmacological intervention on endothelin, vasopressin, and natriuretic peptide systems using, for instance, neuroendocrine analogues might be a new approach to the therapy of dysautonomic syndromes.

### Study strengths and limitations

In spite of the fact that our material provides data from a large number of patients with a recent history of syncope, no control subjects were included, as the approval of Regional Ethical Review Board did not allow inclusion of asymptomatic controls. However, HUT-related hemodynamic responses in the reference (first) quartiles and median values were very similar to those observed in normal populations and among controls [32]. Further, neurohormones were not measured in the late phase of HUT and/or after syncope. Although this information could give more detailed information about neuroendocrine responses during syncope, it is unlikely that it would change the main conclusions of this study, where the aim was to investigate the markers of haemodynamic distress before circulatory collapse, possibly allowing development of therapeutic strategies to employ at the onset of upright posture. It is necessary to emphasize that these were patients referred to a syncope clinic and, thus, included some with hemodynamic characteristics of POTS but in whom the final HUT outcome was syncope. This is well recognized by Grubb who has estimated that around 30% of patients with POTS also have syncope [33].

The assays used in our study detected more stable fragments of ANP, ET-1, and vasopressin with a relatively longer half-life. However, for the detection of catecholamines and renin, standard laboratory assays available at the study site were used instead.

### Major conclusions

- Resting CT-proET-1 and CT-proAVP are increased in orthostatic hypotension, while resting MR-proANP is decreased in postural tachycardia.
- Vasopressin and epinephrine are released by abrupt hypotension, whereas increase in both epinephrine and norepinephrine parallel postural tachycardia.
- These observations may permit better understanding of and therapy in orthostatic intolerance syndromes.

## Supporting Information

S1 Table (DOCX)

S2 Table (DOCX)

S3 Table (DOCX)

S4 Table (DOCX)
