# Supplementary material for: Orthostatic Changes in Hemodynamics and Cardiovascular Biomarkers in Dysautonomic Patients
Source: PLoS One. 2015 Jun 8;10(6):e0128962. doi: 10.1371/journal.pone.0128962 (PMC4460014; doi:10.1371/journal.pone.0128962)
Supplement: S1 Table — (DOCX) [file pone.0128962.s001.docx]

**S1 Table**

Neurohormone concentrations in supine position and their changes after 3 minutes of HUT stratified by quartiles of SBP change after 3 minutes of HUT.

|  | **Quartiles of SBP change after 3 minutes of HUT** | | | |  |
| --- | --- | --- | --- | --- | --- |
| **Neurohormones**  Median (interquartile range) | **Q1**  **Increase**  **> 5 mmHg** | **Q2**  **Increase**  **+5 to -1 mmHg** | **Q3**  **Decrease**  **from 1 to 10 mmHg** | **Q4**  **Decrease**  **> 10 mmHg** | p-value* |
| MR-proANP supine (ρm/L) | 64.8  (43.9-120.4) | 65.1  (45.5-93.1) | 66.1  (48.8-118.3) | 113.9  (64.6-181.3) | <0.001 |
| CT-proET-1 supine (ρm/L) | 54.1  (44.3-64.5) | 50.3  (50.3-75.8) | 51.0  (41.5-62.7) | 62.1  (52.0-74.9) | <0.001 |
| CT-proAVP supine (ρm/L) | 6.75  (4.02-9.84) | 5.66  (3.24-10.03) | 7.34  (4.47-11.95) | 8.65  (4.66-15.00) | <0.001 |
| Renin supine  (mU/L) | 12  (8-20) | 13  (8-20) | 14  (9-27.5) | 14  (9-25.8) | 0.041 |
| Epinephrine supine (nmol/L) | 0.13  (0.08-0.23) | 0.14  (0.09-0.21) | 0.13  (0.08-0.19) | 0.15  (0.09-0.24) | 0.51 |
| Norepinephrine supine (nmol/L) | 2.10  (1.43-2.88) | 2.00  (1.30-2.85) | 2.00  (1.20-2.70) | 2.20  (1.43-3.10) | 0.49 |
| Delta MR-proANP (ρm/L) | 1.8  (-0.6-5.4) | 2.1  (-0.4-4.9) | 2.0  (-0.5-5.3) | 1.8  (-4.0-5.1) | 0.80 |
| Delta CT-proET-1 (ρm/L) | 0.4  (-2.8-2.2) | 0.1  (-2.6-2.1) | 0.0  (-2.8-1.9) | 0.1  (-4.0-2.6) | 0.98 |
| Delta CT-proAVP (ρm/L) | 0.02  (-1.01-1.26) | -0.09  (-1.48-0.51) | 0.14  (-0.83-1.15) | 0.06  (-1.00-1.32) | 0.10 |
| Delta renin  (mU/L) | 0.0  (-1.0-1.0) | 0.0  (-1.0-1.0) | 0.0  (-1.0-1.0) | 0.0  (-1.0-1.0) | 0.95 |
| Delta epinephrine (nmol/L) | 0.05  (0.01-0.11) | 0.05  (0.01-0.12) | 0.05  (0.01-0.12) | 0.05  (0.00-0.14) | 0.96 |
| Delta norepinephrine (nmol/L) | 1.1  (0.8-1.4) | 1.0  (0.7-1.5) | 1.1  (0.7-1.65) | 0.9  (0.5-1.6) | 0.40 |

HUT, head-up tilt test; SBP, systolic blood pressure; MR-proANP, midregional fragment of pro-atrial natriuretic peptide; CT-proET-1, C-terminal endothelin-1 precursor fragment; CT-proAVP, C-terminal pro-arginine vasopressin; *according to Kruskal-Wallis test for differences between groups.
