# Supplementary material for: Orthostatic Changes in Hemodynamics and Cardiovascular Biomarkers in Dysautonomic Patients
Source: PLoS One. 2015 Jun 8;10(6):e0128962. doi: 10.1371/journal.pone.0128962 (PMC4460014; doi:10.1371/journal.pone.0128962)
Supplement: S2 Table — (DOCX) [file pone.0128962.s002.docx]

**S2 Table**

Neurohormone concentrations in supine position and their changes after 3 minutes of HUT stratified by quartiles of maximal SBP change during HUT.

|  | **Quartiles of maximal SBP change during HUT** | | | |  |
| --- | --- | --- | --- | --- | --- |
| **Neurohormones**  Median (interquartile range) | **Q1**  **Decrease**  **< 7 mmHg** | **Q2**  **Decrease**  **from 7 to 16 mmHg** | **Q3**  **Decrease**  **from 16 to 30 mmHg** | **Q4**  **Decrease**  **> 30 mmHg** | p-value* |
| MR-proANP supine (ρm/L) | 63.4  (43.8-120.4) | 62.1  (41.5-93.1) | 67.6  (46.2-122.3) | 116.4  (67.4-189.9) | <0.001 |
| CT-proET-1 supine (ρm/L) | 50.8  (42.5-58.4) | 49.1  (41.2-62.4) | 52.3  (43.4-66.2) | 63.0  (52.7-74.9) | <0.001 |
| CT-proAVP supine (ρm/L) | 6.81  (3.79-10.33) | 6.19  (3.53-10.22) | 7.01  (3.75-11.48) | 8.12  (5.01-14.56) | 0.011 |
| Renin supine  (mU/L) | 12  (7-19) | 14  (8-22) | 13  (9-21) | 14.5  (9-31) | 0.04 |
| Epinephrine supine (nmol/L) | 0.13  (0.08-0.21) | 0.12  (0.08-0.19) | 0.14  (0.09-0.24) | 0.15  (0.09-0.24) | 0.15 |
| Norepinephrine supine (nmol/L) | 1.90  (1.23-2.88) | 1.80  (1.20-2.85) | 2.10  (1.50-2.70) | 2.40  (1.60-3.10) | <0.001 |
| Delta MR-proANP (ρm/L) | 1.9  (-0.6-5.1) | 1.9  (0.1-4.8) | 1.9  (-1.7-5.3) | 1.8  (-3.1-5.4) | 0.98 |
| Delta CT-proET-1 (ρm/L) | 0.4  (-2.8-2.1) | -0.1  (-2.7-1.9) | 0.2  (-2.9-2.9) | 0.0  (-2.9-2.1) | 0.63 |
| Delta CT-proAVP (ρm/L) | -0.08  (-1.10-1.03) | 0.00  (-1.23-1.11) | 0.13  (-0.76-1.58) | 0.00  (-1.04-0.60) | 0.26 |
| Delta renin  (mU/L) | 0.0  (-1.0-1.0) | 0.0  (-1.0-1.0) | 0.0  (-1.0-1.0) | 0.0  (-1.0-1.0) | 0.54 |
| Delta epinephrine (nmol/L) | 0.06  (0.02-0.13) | 0.05  (0.02-0.12) | 0.05  (0.01-0.12) | 0.04  (0.00-0.11) | 0.24 |
| Delta norepinephrine (nmol/L) | 1.0  (0.7-1.5) | 0.9  (0.6-1.4) | 1.1  (0.8-1.6) | 1.1  (0.6-1.8) | 0.09 |

HUT, head-up tilt test; SBP, systolic blood pressure; MR-proANP, midregional fragment of pro-atrial natriuretic peptide; CT-proET-1, C-terminal endothelin-1 precursor fragment; CT-proAVP, C-terminal pro-arginine vasopressin; *according to Kruskal-Wallis test for differences between groups.
