# Supplementary material for: Orthostatic Changes in Hemodynamics and Cardiovascular Biomarkers in Dysautonomic Patients
Source: PLoS One. 2015 Jun 8;10(6):e0128962. doi: 10.1371/journal.pone.0128962 (PMC4460014; doi:10.1371/journal.pone.0128962)
Supplement: S3 Table — (DOCX) [file pone.0128962.s003.docx]

**S3 Table**

Neurohormone concentrations in supine position and their changes after 3 minutes of HUT stratified by quartiles of heart rate change after 3 minutes of HUT.

|  | **Quartiles of heart rate change after 3 minutes of HUT** | | | |  |
| --- | --- | --- | --- | --- | --- |
| **Neurohormones**  Median (interquartile range) | **Q1**  **Increase**  **< 3 bpm** | **Q2**  **Increase**  **from 3 to 9 bpm** | **Q3**  **Increase**  **from 9 to 15 bpm** | **Q4**  **Increase**  **> 15 bpm** | p-value* |
| MR-proANP supine (ρm/L) | 115.8  (72.7-186.7) | 88.3  (58.2-140.3) | 62.7  (44.9-105.7) | 45.7  (35.5-66.0) | <0.001 |
| CT-proET-1 supine (ρm/L) | 59.5  (50.1-74.6) | 56.4  (46.2-72.4) | 53.4  (44.1-65.1) | 46.0  (38.2-56.2) | <0.001 |
| CT-proAVP supine (ρm/L) | 8.63  (4.09-14.27) | 6.94  (3.84-11.57) | 7.01  (4.14-10.53) | 5.91  (3.43-9.37) | 0.014 |
| Renin supine  (mU/L) | 13  (7-21) | 14  (8-20) | 14  (9-25) | 14  (9-22) | 0.21 |
| Epinephrine supine (nmol/L) | 0.16  (0.11-0.24) | 0.14  (0.08-0.23) | 0.13  (0.09-0.22) | 0.12  (0.07-0.18) | 0.001 |
| Norepinephrine supine (nmol/L) | 2.45  (1.60-3.13) | 2.20  (1.40-3.00) | 2.00  (1.40-2.70) | 1.60  (1.10-2.30) | <0.001 |
| Delta MR-proANP (ρm/L) | 2.3  (-2.0-6.7) | 2.1  (-0.6-5.8) | 1.4  (-1.3-4.2) | 1.7  (-0.3-3.6) | 0.08 |
| Delta CT-proET-1 (ρm/L) | 0.4  (-2.9-2.9) | 0.0  (-2.6-2.1) | -0.4  (-3.6-2.0) | 0.4  (-2.3-1.6) | 0.24 |
| Delta CT-proAVP (ρm/L) | -0.11  (-1.32-0.80) | -0.09  (-1.00-0.72) | 0.02  (-0.82-0.80) | 0.25  (-0.68-1.67) | 0.08 |
| Delta renin  (mU/L) | 0.0  (-1.0-1.0) | 0.0  (-1.0-1.0) | 0.0  (-1.0-1.0) | 0.0  (-1.0-1.0) | 0.47 |
| Delta epinephrine (nmol/L) | 0.03  (0.00-0.09) | 0.05  (0.01-0.11) | 0.05  (0.02-0.12) | 0.08  (0.03-0.19) | <0.001 |
| Delta norepinephrine (nmol/L) | 1.0  (0.7-1.4) | 1.0  (0.6-1.3) | 1.1  (0.7-1.7) | 1.3  (0.8-1.8) | 0.001 |

HUT, head-up tilt test; SBP, systolic blood pressure; MR-proANP, midregional fragment of pro-atrial natriuretic peptide; CT-proET-1, C-terminal endothelin-1 precursor fragment; CT-proAVP, C-terminal pro-arginine vasopressin; *according to Kruskal-Wallis test for differences between groups
