# Supplementary material for: Orthostatic Changes in Hemodynamics and Cardiovascular Biomarkers in Dysautonomic Patients
Source: PLoS One. 2015 Jun 8;10(6):e0128962. doi: 10.1371/journal.pone.0128962 (PMC4460014; doi:10.1371/journal.pone.0128962)
Supplement: S4 Table — (DOCX) [file pone.0128962.s004.docx]

**S4 Table**

Neurohormone concentrations in supine position and their changes after 3 minutes of HUT stratified by quartiles of maximal heart rate change during HUT.

|  | **Quartiles of maximal heart rate change during HUT** | | | |  |
| --- | --- | --- | --- | --- | --- |
| **Neurohormones**  Median (interquartile range) | **Q1**  **Increase**  **< 5 bpm** | **Q2**  **Increase**  **from 5 to 11 bpm** | **Q3**  **Increase**  **from 11 to 21 bpm** | **Q4**  **Increase**  **> 21 bpm** | p-value* |
| MR-proANP supine (ρm/L) | 121.0  (81.1-200.6) | 78.6  (52.5-130.7) | 65.9  (45.4-112.9) | 50.3  (37.3-66.4) | <0.001 |
| CT-proET-1 supine (ρm/L) | 60.4  (50.9-74.9) | 56.2  (46.1-66.6) | 55.1  (44.4-65.9) | 45.3  (40.1-54.8) | <0.001 |
| CT-proAVP supine (ρm/L) | 8.03  (4.24-14.10) | 7.00  (3.77-11.06) | 7.39  (3.56-12.13) | 5.89  (3.60-9.10) | 0.003 |
| Renin supine  (mU/L) | 11.5  (7-21) | 14  (8-21) | 14  (8-23) | 14  (9-22) | 0.33 |
| Epinephrine supine (nmol/L) | 0.15  (0.10-0.24) | 0.15  (0.09-0.23) | 0.14  (0.09-0.23) | 0.11  (0.06-0.16) | <0.001 |
| Norepinephrine supine (nmol/L) | 2.50  (1.60-3.40) | 2.20  (1.50-2.80) | 2.10  (1.38-2.75) | 1.60  (1.12-2.38) | <0.001 |
| Delta MR-proANP (ρm/L) | 2.0  (-2.7-6.6) | 1.6  (-0.9-5.1) | 1.8  (-1.0-4.8) | 2.1  (0.6-4.4) | 0.55 |
| Delta CT-proET-1 (ρm/L) | 0.1  (-3.5-2.6) | 0.2  (-3.1-2.1) | 0.0  (-3.3-2.6) | 0.4  (-2.0-2.0) | 0.80 |
| Delta CT-proAVP (ρm/L) | 0.15  (-1.19-0.77) | -0.09  (-1.19-0.85) | 0.00  (-0.88-0.79) | 0.15  (-0.68-1.60) | 0.15 |
| Delta renin  (mU/L) | 0.0  (-1.0-1.0) | 0.0  (-1.0-1.0) | 0.0  (-1.0-1.0) | 0.0  (-1.0-1.0) | 0.13 |
| Delta epinephrine (nmol/L) | 0.03  (0.00-0.09) | 0.06  (0.01-0.12) | 0.05  (0.02-0.11) | 0.09  (0.02-0.18) | <0.001 |
| Delta norepinephrine (nmol/L) | 1.0  (0.6-1.3) | 1.0  (0.7-1.5) | 1.1  (0.7-1.6) | 1.2  (0.7-1.8) | 0.044 |

HUT, head-up tilt test; SBP, systolic blood pressure; MR-proANP, midregional fragment of pro-atrial natriuretic peptide; CT-proET-1, C-terminal endothelin-1 precursor fragment; CT-proAVP, C-terminal pro-arginine vasopressin; *according to Kruskal-Wallis test for differences between groups.
